# Protocol for efficient CRISPR/AAV-mediated genome editing and erythroid differentiation of human hematopoietic stem and progenitor cells

**DOI:** 10.1016/j.xpro.2025.104018

**Published:** 2025-08-06

**Authors:** Devesh Sharma, Roshani Sinha, Benjamin J. Lesch, M. Kyle Cromer

**Affiliations:** 1 Department of Surgery, University of California, San Francisco, San Francisco, CA 94143, USA; 2 Eli & Edythe Broad Center for Regeneration Medicine, University of California, San Francisco, San Francisco, CA 94143, USA; 3 Department of Bioengineering & Therapeutic Sciences, University of California, San Francisco, San Francisco, CA 94158, USA

**Keywords:** Cell Biology, Cell culture, Flow Cytometry, Molecular Biology, CRISPR, Stem Cells, Cell Differentiation, Biotechnology and bioengineering

## Abstract

Here, we present a protocol for genome editing in human hematopoietic stem and progenitor cells (HSPCs) using CRISPR-Cas9 ribonucleoproteins and adeno-associated virus (AAV)-mediated homology-directed repair. We describe steps for AAV production, purification, and titration; HSPC thawing and culture; genome editing; and quantification of editing frequencies. We then detail procedures for erythroid differentiation assays. This protocol ensures high editing efficiency while maintaining cell viability and engraftment potential.

For complete details on the use and execution of this protocol, please refer to Chu et al.^1^

## Highlights

• Step-by-step guide for production and purification of DNA repair templates
• Protocol for high-efficiency, precision genome editing in hematopoietic stem cells
• Techniques for *in vitro* erythroid differentiation and flow cytometry analysis

## Abstract (editor highlights)

Publisher’s note: Undertaking any experimental protocol requires adherence to local institutional guidelines for laboratory safety and ethics.

## Graphical abstract

**Figure:**
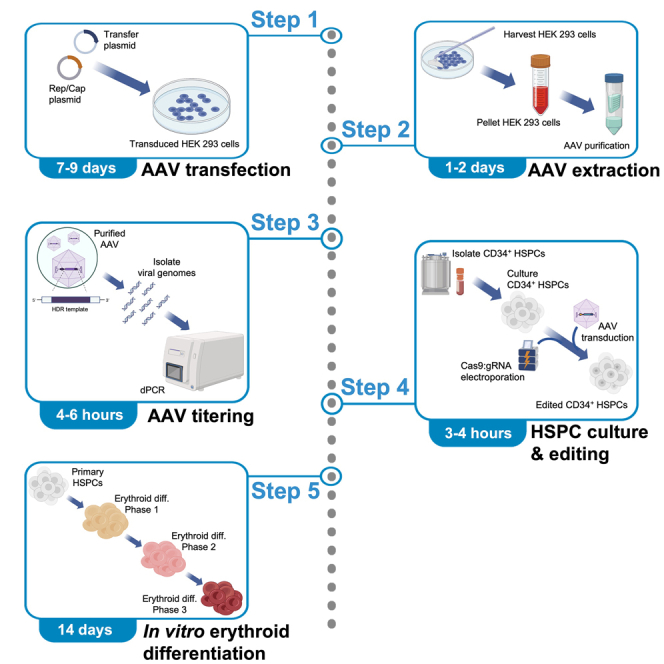

## Before you begin

### Experimental design considerations

**Timing: 1–2 weeks**

1. Thaw and expand HEK 293T cells before use.

***Note:*** Healthy and low passage HEK 293T cells are critical for a high yield of AAV. The HEK 293T cells used for AAV production are maintained below 80% confluence and passaged less than 15 times. After thawing, HEK 293T cells should be kept in culture for at least one passage before seeding for AAV production.

2. Design Cas9 guide RNAs (gRNAs) surrounding the desired cleavage site using CHOPCHOP (https://chopchop.cbu.uib.no/).^2^
3. Screen gRNAs for potential off-target editing sites using COSMID (https://crispr.bme.gatech.edu/).^3^

***Note:*** Our recommendation is testing at least 5 distinct gRNAs if targeting a novel region of the genome. When doing so, try to select gRNAs with no COSMID-nominated off-target sites in coding regions of the genome with score <5.5.^4^

4. Validate gRNAs by delivering Cas9 RNP via electroporation to HSPCs (refer to protocol below).

***Note:*** Validate indel frequency by Sanger sequencing with comparison to negative control. We recommend using TIDE (https://tide.nki.nl/) to quantify indel frequencies,^5^ and ideally identify a gRNA with >75% indel frequency for use in downstream editing experiments (either for cleavage alone or mediating HDR).

5. Design donor templates with 200–1,000 bp homology arms to maximize homology-directed repair (HDR) efficiency.

***Note:*** Maintain AAV genome length below 4.5 kb (including inverse terminal repeats (ITRs)) due to packaging constraints.

6. Obtain accurate AAV vector genome titration using digital PCR (dPCR).

***Note:*** Order validated ITR-specific primer/probes^6^ or design custom primer/probes with primers corresponding to the HDR template packaged between the AAV ITRs.

7. Evaluate HDR efficiency.

***Note:*** Design a primer/probe set for dPCR with one primer and the detection probe binding inside the inserted sequence and the other primer binding outside the homology arm to specifically detect successful integration.

8. Ensure availability of human CD34^+^ HSPCs.

***Note:*** These can be isolated from umbilical cord blood, bone marrow aspirates, or mobilized peripheral blood mononuclear cells (PBMCs). If isolating from any of these sources, perform magnetic bead enrichment to obtain a high-purity CD34^+^ population. The quality and enrichment of HSPCs are critical for reproducibility in downstream genome editing and differentiation steps.

9. For transplanting HSPCs into immuno-deficient mice, we recommend maintaining cells in hypoxic conditions (5% O_2_, 5% CO_2_, 37°C) during *ex vivo* culture and expansion.

***Note:*** If only performing *ex vivo* HSPC editing and/or erythroid differentiation, then hypoxic conditions are not necessary.

## Key resources table

**Table:** 

REAGENT or RESOURCE	SOURCE	IDENTIFIER
**Antibodies**		

Human CD34 - APC. Working dilution 1:50	BD Biosciences	561
Human CD45 - V450. Working dilution 1:50	BioLegend	HI30
Human CD71 - PE-Cy7. Working dilution 1:50	Affymetrix	OKT9
Human CD235a (GPA) - PE. Working dilution 1:50	BD Biosciences	GA-R2
Human CD271 (NGFR) - PE. Working dilution 1:50	BioLegend	ME20.4
Human CD36 - APC. Working dilution 1:50	BioLegend	5-271
DRAQ5. Working dilution 1:200	BioLegend	424101
Viability dye. Working dilution 1:100	Cytek Biosciences	13-0865-T100

**Bacterial and virus strains**		

Stable competent *E. coli*	New England Biolabs	C3040I
AAV serotype 6 rep/cap production plasmid (pDGM6)	GenScript (plasmid originally from David Russell)	Custom industrial grade order

**Biological samples**		

Heat-inactivated human type AB serum	Sigma-Aldrich	H3667
Human plasma	Innovative Research	IPLAWBCATNAC

**Chemicals, peptides, and recombinant proteins**		

*S. pyogenes* high-fidelity Cas9	Integrated DNA Technologies, Aldevron	1081058, 9212
Human SCF	PeproTech	300-07
Human TPO	PeproTech	300-18
Human FLT3L	PeproTech	300-19
Human IL6	PeproTech	200-06
Human IL3	PeproTech	200-03
Human EPO	PeproTech	100-64
Human insulin	Sigma-Aldrich	91077C
Human holo-transferrin	Sigma-Aldrich	T0665
Heparin sodium salt	Sigma-Aldrich	H3393
Dimethyl sulfoxide (DMSO)	Sigma-Aldrich	67-68-5
Phosphate-buffered saline (PBS)	Thermo Fisher Scientific	10010001
Penicillin-streptomycin (10,000 U/mL; 10,000 μg/mL)	Thermo Fisher Scientific	15140148
L-glutamine (200 mM)	Thermo Fisher Scientific	35050061
Polyethylenimine (PEI)	Polysciences	23966
UM171	MedChemExpress	HY-12878
SFEM II medium	STEMCELL Technologies	09655
DMEM (high glucose) medium	Thermo Fisher Scientific	11965092
Opti-MEM medium	Thermo Fisher Scientific	31985062
RPMI 1640 medium	Thermo Fisher Scientific	11875093
Heat-inactivated fetal bovine serum (FBS)	Thermo Fisher Scientific	A5209501
Ethylenediaminetetraacetic acid (EDTA) disodium salt dihydrate	Sigma-Aldrich	E4884
HEPES (1.0 M)	Thermo Fisher Scientific	J60712.AP
PBS (pH 7.2)	Thermo Fisher Scientific	20012027
Sodium pyruvate	Thermo Fisher Scientific	11360070
Non-essential amino acids (100×)	Thermo Fisher Scientific	11140050
DNase	Sigma-Aldrich	9003-98-9
TrypLE Express (1×)	Thermo Fisher Scientific	12604013
*Rev*IT AAV production enhancer	Mirus Bio	MIR 8000

**Experimental models: Organisms/strains**		

Human CD34^+^-enriched HSPCs derived from umbilical cord blood, bone marrow aspirate, or Plerixafor and/or G-CSF-mobilized peripheral blood	STEMCELL Technologies, AllCells, Fred Hutchinson Cancer Center Hematology Core	Various
HEK 293T cells	ATCC	CRL-3216

**Oligonucleotides**		

Custom Cas9 gRNA	Synthego	Custom gRNA
dPCR primers & probes	Integrated DNA Technologies	Custom probe-based assays

**Recombinant DNA**		

AAV6 DNA repair templates	Cloned in-house & purified using Takara Bio AAVpro purification kit	6675

**Other**		

Endotoxin-free plasmid purification kit	QIAGEN	12362
gDNA extraction kit	QIAGEN	69504
Bambanker freezing media	Bulldog Bio	BB01
TRIzol reagent	Thermo Fisher Scientific	15596026
Cresyl blue solution	Sigma-Aldrich	1013840100
QIAcuity probe PCR kit	QIAGEN	250103
QIAcuity Nanoplate, 8.5k 24-well	QIAGEN	250011
QuickExtract DNA extraction solution (QE)	Lucigen	76081-768
Phusion Green PCR master mix	Thermo Fisher Scientific	F632S
DNA ladder	Thermo Fisher Scientific	10787018
Midori Green DNA stain	Bulldog Bio	MG06
Tris-acetate-EDTA (TAE) buffer	Thermo Fisher Scientific	B49

## Materials and equipment

**Table:** HEK 293T culture media

Reagent (stock concentration)	Volume	Final concentration
DMEM (high glucose) medium	425 mL	N/A
Heat-inactivated fetal bovine serum (FBS)	50 mL	10% volume
Penicillin-streptomycin (10,000 U/mL; 10,000 μg/mL)	5 mL	1% volume
L-glutamine (200 mM)	5 mL	1% volume
HEPES (1.0 M)	5 mL	1% volume
Sodium pyruvate (100 mM)	5 mL	1% volume
Non-essential amino acids (100×)	5 mL	1% volume
Total	500 mL	N/A

Mix well in the hood and sterile filter with 0.45 μm filters. Store at 4°C for up to 1 month.

**Table:** PEI stock solution

Reagent (stock concentration)	Volume	Final concentration
Polyethylenimine (PEI)	1 gm	N/A
MilliQ water	10 mL	N/A
Hydrochloric acid (HCl)	As needed	To adjust pH to ∼2
Sodium hydroxide (NaOH)	As needed	To adjust pH to ∼7
Total	10 mL	1 gm/mL

Add PEI to MilliQ water (1 mg/mL final concentration) and stir on a magnetic stir plate. Adjust pH to ∼2 using concentrated HCl to fully dissolve the PEI. Once dissolved, adjust pH back to 7.0 using NaOH. Sterile filter through a 0.22 μm filter, aliquot, and store at −80°C for up to 1 year.

**Table:** AAV transfection media

Reagent (stock concentration)	Volume	Final concentration
Opti-MEM medium	435 mL	N/A
Heat-inactivated fetal bovine serum (FBS)	50 mL	5% volume
Non-essential amino acids (100×)	5 mL	1% volume
Sodium pyruvate (100 mM)	5 mL	1% volume
L-glutamine (200 mM)	5 mL	1% volume
Total	500 mL	N/A

Mix well in the hood and sterile filter with 0.45 μm filters. Store at 4°C for up to 1 month.

**Table:** HSPC thaw media

Reagent (stock concentration)	Volume	Final concentration
RPMI 1640 medium	340 mL	N/A
Penicillin-streptomycin (10,000 U/mL; 10,000 μg/mL)	5 mL	1% volume
Heat-inactivated fetal bovine serum (FBS)	150 mL	30% volume
DNase (1 mg/mL)	5 mL	1% volume
Total	500 mL	N/A

Mix well in the hood and sterile filter with 0.45 μm filters. Store at −20°C for up to 1 month.

**Table:** HSPC culture media

Reagent (stock concentration)	Volume	Final concentration
SFEM II medium	25 mL	N/A
Penicillin-streptomycin (10,000 U/mL; 10,000 μg/mL)	50 μL	20 U/mL; 20 mg/mL
SCF (100 μg/mL)	2.5 μL	100 ng/mL
TPO (100 μg/mL)	2.5 μL	100 ng/mL
FLT3L (100 μg/mL)	2.5 μL	100 ng/mL
IL6 (100 μg/mL)	2.5 μL	100 ng/mL
Total	25.06 mL	N/A

Mix well in the hood and sterile filter with 0.45 μm filters. Store at 4°C for up to 1 week.

**Table:** Flow cytometry buffer

Reagent	Volume	Final concentration
PBS (pH 7.2)	470 mL	N/A
Heat-inactivated fetal bovine serum (FBS)	25 mL	5% volume
Ethylenediaminetetraacetic acid (EDTA) disodium salt dihydrate	5 mL	2 mM
Total	500 mL	N/A

Mix well in the hood and sterile filter with 0.45 μm filters. Store at 4°C for up to 2 weeks.

**Table:** Erythroid differentiation media

Reagent (stock concentration)	Day 0–6 (phase 1)		Day 7–10 (phase 2)		Day 11–14 (phase 3)	
	Volume	Final conc.	Volume	Final conc.	Volume	Final conc.
SFEM II media	25 mL	N/A	25 mL	N/A	25 mL	N/A
Penicillin-streptomycin (10,000 U/mL; 10,000 μg/mL)	250 μL	100 U/mL; 100 μg/mL	250 μL	100 U/mL; 100 μg/mL	250 μL	100 U/mL; 100 μg/mL
SCF (100 μg/mL)	2.5 μL	10 ng/mL	2.5 μL	10 ng/mL	–	–
IL3 (10 μg/mL)	2.5 μL	1 ng/mL	–	–	–	–
EPO (1,000 U/mL)	75 μL	3 U/mL	75 μL	3 U/mL	75 μL	3 U/mL
Transferrin (50 mg/mL)	100 μL	200 μg/mL	100 μL	200 μg/mL	500 μL	1 mg/mL
Heat-inactivated human type AB serum	750 μL	3% volume	750 μL	3% volume	750 μL	3% volume
Human plasma	500 μL	2% volume	500 μL	2% volume	500 μL	2% volume
Insulin (10 mg/mL)	25 μL	10 μg/mL	25 μL	10 μg/mL	25 μL	10 μg/mL
Heparin (7,000 U/mL)	10.7 μL	3 U/mL	10.7 μL	3 U/mL	10.7 μL	3 U/mL

Mix well in the hood and sterile filter with 0.45 μm filters. Store at −20°C for up to 3 months.

## Step-by-step method details

### AAV production in HEK 293T cells

**Timing: 7–9 days**

**Timing: 4–5 days (for step 1)**

This section describes the detailed steps for culturing HEK 293T cells and producing recombinant AAV vectors using a PEI-based transfection method followed by purification using the Takara AAVpro Purification Kit (Midi). The workflow emphasizes best practices for ensuring high transfection efficiency and virus recovery, using standard lab equipment and reagents.

1. Culturing and expanding HEK 293T cells.
  ***Note:*** This step involves thawing and expanding HEK 293T cells under optimal culture conditions to maintain their health and transfection competence.
  a. Thaw a low-passage vial of cryopreserved HEK 293T cells in a 37°C water bath.
  b. Transfer the thawed suspension to a 15 mL conical tube containing 10 mL of pre-warmed HEK 293T Culture Media in a slow swirling motion.
  c. Centrifuge at 200 × *g* for 5 min at 20°C–25°C.
  d. Aspirate the supernatant and resuspend the cell pellet in fresh HEK 293T Culture Media.
  e. Seed 2 million cells in a T225 flask with 30–45 mL HEK 293T Culture Media.
  f. Incubate at 37°C in a humidified 5% CO_2_ incubator.
    ***Optional:*** For enhanced AAV production, consider using commercially available HEK-derived cell lines optimized for high-titer AAV yield, such as Takara's AAVpro 293T cells. These cells are specifically selected for consistency and efficiency in AAV vector production.
    ***Note:* 1**. Monitor confluency daily. Cells should reach 40%–50% confluency in ∼3 days. Avoid surpassing 60% confluency to maintain optimal morphology.
    ***Note:* 2.** Passage routinely at 1:7 every 2 days or 1:20 every 3 days, depending on growth rate.
    **CRITICAL:** Properly maintained HEK 293T cells will display distinct cell margins. Discard cultures that appear clumped or fused.

#### Seeding for transfection (day −1)

**Timing: 2 h**

2. Prepare HEK 293T cells in AAV Transfection Media.
  a. Gently rinse flask of HEK 293T cells (ideally 40% confluence) with 10 mL PBS and aspirate.
  b. Add 5 mL TrypLE Express and incubate 3–5 min.
  c. Neutralize with 10 mL AAV Transfection Media and transfer to a 50 mL conical tube.
  d. Count cells using a hemocytometer or automated cell counter.
  e. Plate 20 million cells in a T225 flask using 45 mL AAV Transfection Media.
  f. Incubate for 8–12 h at 37°C.

***Note:*** Cells should be ∼90% confluent the next morning, with visible intercellular margins and no overgrowth.

#### Transfection (day 0)

**Timing: 2–3 h, followed by 48–72 h incubation**

3. Preparation and delivery of transfection mix.
  a. Warm plain Opti-MEM media (without additives) to 20°C–25°C.
  b. For each condition (T225 flask), prepare the transfection mix in the following order:
    i. Add 2 mL of Opti-MEM media to a sterile tube.
    ii. Add 6 μg of transfer plasmid (i.e., the custom DNA template for mediating HDR; grown in stable competent *E. coli* and prepped with commercial endotoxin-free plasmid purification kit).
    iii. Add 22 μg of pDGM6 (rep/cap and helper in one plasmid).
    iv. Add *Rev*IT AAV production enhancer to a final concentration of 1 μL/mL.
    v. Add 112 μL PEI (1 mg/mL) as the final reagent.
  c. Gently invert the tube after addition of each item to mix without introducing bubbles and ensure no precipitates form.
  d. Incubate transfection mix at 20°C–25°C for 15 min to allow complex formation.
  e. Add 2 mL of the transfection mix dropwise per T225 flask. Gently rock flasks side-to-side to ensure even distribution across the cell surface.
  f. Incubate cells for 48–72 h at 37°C.

***Note:* 1.** At 24 h post-transfection you may observe morphology under a microscope—successful transfection typically causes rounding and partial detachment of cells.

***Note:* 2.** Media change is not required unless cytotoxicity is observed. Transfection can be optimized by testing different commercially available AAV enhancer combinations.

#### AAV harvest (days 2–3)

**Timing: ∼2 h**

4. Recovery of AAV-producing cells for downstream AAV extraction.
  a. Harvest HEK 293T cells from plate by pipetting media up and down or using a cell scraper to dislodge cells into media.
    ***Optional:*** If scraping T225 flasks is challenging, consider using an equivalent number of 15 cm^2^ dishes to facilitate easier cell collection.
  b. Transfer cell suspensions into 50 mL Falcon tubes.
  c. Centrifuge at 2,000 × *g* for 10 min at 4°C.
  d. Carefully discard supernatant.
    ***Note:*** This protocol focuses on harvesting cell-associated AAV particles. However, some protocols retain the supernatant and achieve comparable titers. Users may adapt based on lab preference and optimization.
  e. Perform a second spin at 2,000 × *g* for 1 min at 4°C and remove residual supernatant.
    **CRITICAL:** Ensure complete removal of supernatant to avoid interference with virus purification.
    **Pause point:** Cell pellets may proceed directly to purification or be frozen at −80°C for processing later.

### AAV purification steps

#### Safety and handling of AAVs

This section outlines the required safety procedures when working with AAV vectors. These vectors can express transgenes *in vivo* and may be hazardous depending on the gene insert. Adhere to all biosafety guidelines and institutional regulations for recombinant DNA work.

5. Wear appropriate personal protective equipment (PPE) including lab coats, gloves, and face protection.
6. Dispose of all AAV-contaminated materials in accordance with institutional biosafety regulations.
7. Decontaminate work surfaces and equipment after handling AAV materials.

#### AAV purification from producer cells using a commercial kit

**Timing: ∼4 h**

This section enables the purification and concentration of AAV particles from HEK 293T cells using a commercial kit, eliminating the need for iodixanol preparation and ultracentrifugation. This approach yields high-titer AAV suitable for *in vitro* applications. However, *in vivo*-grade AAV may need to be purified in a more thorough and systematic process.

***Note:*** When using frozen cell pellets, thaw cell pellets at 20°C–25°C. Loosen pellet by thoroughly vortexing.

**CRITICAL:** Make sure to have a homogenous solution. Cell clumps reduce purification efficiency.

8. Downstream processing and purification of AAV vectors.
  a. Add 2 mL of AAV Extraction Solution A. Vortex for 15 s until fully resuspended.
  b. Incubate at 20°C–25°C for 5 min and vortex again for 15 s.
  c. Centrifuge at 8,000 × *g* for 10 min at 4°C.
    ***Optional:*** Repeat steps 8a–8c to improve virus yield.
  d. Transfer the supernatant to a sterile centrifuge tube.
  e. Add 1/10^th^ volume of AAV Extraction Solution B.
    ***Note:*** Solution typically turns pink after adding Extraction Solution B. This does not affect performance.
    **Pause point:** Virus suspension may either proceed immediately to the next step or be stored at −80°C. If frozen, thaw at 37°C before use.

#### AAV vector purification using kit

9. Add 1/100^th^ volume of Cryonase cold-active nuclease to the virus suspension.
10. Incubate at 37°C for 1 h.
11. Add 1/10^th^ volume of Precipitator A, vortex for 10 s, incubate at 37°C for 30 min, and vortex again.

***Note:*** If any precipitate forms in Precipitator A, dissolve by warming to 37°C before use.

12. Add 1/20^th^ volume of Precipitator B, vortex for 10 s.
13. Centrifuge at 5,000–9,000 × *g* for 5 min at 4°C.
14. Filter the supernatant using a Millex-HV 0.45 μm filter.
15. Transfer the solution to an Amicon Ultra-4 (100 kDa).
16. Centrifuge at 2,000 × *g* for 5 min at 15°C. Confirm volume is <0.4 mL.
17. Discard flow through.
18. Add 1 mL of Suspension Buffer to the filter device and mix by pipetting.
19. Centrifuge at 2,000 × *g* for 5 min at 15°C. Confirm volume is <0.4 mL.

***Note:*** Spin filters are designed with a minimal hold-up volume which prevents them from spinning down to 0 mL. It is therefore acceptable to centrifuge them until only the hold-up volume (typically 25–50 μL) remains.

20. Repeat buffer exchange until a total of 5 mL of Suspension Buffer has been passed through the filter. Discard final flow through.
21. Mix concentrated AAV by pipetting and transfer to a clean microcentrifuge tube.

**CRITICAL:** Maintain sterility throughout the procedure to avoid contamination. Do not exceed recommended centrifugal forces.

**Pause point:** Concentrated AAV can be stored at −80°C for long-term use.

### Titration of AAV vector genomes by digital PCR

**Timing: 4 h**

**Timing: 20 min (for step 22)**

Accurate quantification of vector genome titers is essential for standardizing recombinant AAV dosing in both preclinical and clinical settings. Here, we present a modified dPCR protocol developed and optimized in-house for streamlined, high-throughput quantification using the QIAcuity Digital PCR System. This adaptation builds upon the standard protocol and includes detailed, step-by-step instructions consistent with our laboratory’s workflow and reagent preferences. This section enables high assay performance with reduced labor compared to traditional droplet digital PCR (ddPCR) and quantitative PCR (qPCR) workflows.

***Note:* 1.** Gene of interest-specific/AAV ITR probe and primers must be designed and synthesized/ordered prior to protocol initiation.

***Note:* 2.** For accurate titering, either commercially validated probes (e.g., for AAV2) or custom designs targeting conserved regions—such as ITRs or transgenes—may be used. For AAV2, validated primers and probes have been designed to target a 62-bp region within the ITR, which have been reported in prior work (Cromer, et al. Nature Medicine, 2021 and others). This design has shown robust amplification across annealing temperatures from 55–64.2°C, with 60°C recommended for optimal performance. Standard primer design principles apply: optimal length (18–30 nt), 30%–70% GC content, and amplicons of 60–150 bp. Due to the palindromic and GC-rich nature of ITRs, empirical optimization—such as performing an annealing temperature gradient—is strongly recommended. Additionally, cassette-specific primer/probe sets may be used as an alternative but require individual validation.

22. AAV pre-treatment using QuickExtract (QE) to extract DNA from the capsid proteins for downstream dPCR reaction.
  a. In a sterile PCR tube, dispense 18 μL of QE.
  b. Thaw the AAV sample on ice. Vortex and spin down the PCR tube.
  c. Add 2 μL of thawed AAV sample to the QE (final 1:10 dilution).
  d. Run the QE reaction on a thermocycler using the following protocol (∼20 min):

**Table:** 

Step	Temperature	Duration
Activation & degradation	65°C	6 min
Cell lysis	100°C	10 min
Infinite hold	12°C	Infinite

#### Preparation of AAV sample dilutions

**Timing: 30 min**

23. Perform serial dilution of QE-treated AAV samples to bring the genomic content within the analytical range suitable for dPCR quantification.
  a. Prepare serial dilutions of the digested sample in nuclease-free water to 1:1K, 1:10K, and 1:100K. To do so, follow this workflow:
    i. Use the 1:10 diluted QE mixed sample and mix 198 μL of water with 2 μL of the 1:10 QE-treated AAV (yields 1:1K dilution).
    ii. Mix 18 μL of water with 2 μL of the 1:1K dilution (yields 1:10K dilution).
    iii. Mix 18 μL of water with 2 μL of the 1:10K dilution (yields 1:100K dilution).

***Note:* 1.** When setting up the dPCR reaction, the above dilutions will be added to the PCR reagents for an additional 1:10 dilution.

***Note:* 2.** Ensure the water used is DNase- and RNase-free for each and every step.

**CRITICAL:** Ensure vortexing thoroughly and spinning down at each dilution step for accurate titers.

#### dPCR master mix preparation

**Timing: 15 min**

24. Prepare and assemble master mix for dPCR reactions according to the following guidelines (scale up based on the number of reactions and dilutions planned).
  **CRITICAL:** Maintain reactions on ice and away from light.
  **Table:** dPCR master mix for one reaction

Reagent	Volume
dPCR Master Mix (QIAGEN)	3.0 μL
ITR Primer/Probe Mix (Integrated DNA Technologies)	0.3 μL
DNase/RNase-free Water	10.2 μL
Diluted AAV Sample	1.5 μL
*Total*	15.0 μL
  Example calculation:
  Calculate the total number of reactions required.
  a. For our midi-scale AAV production, yields, and concentrations that we obtain, we recommend running both 1:100K and 1:1M final dilutions per AAV sample, each in duplicate.
  b. Include 2 no template controls (NTCs).
  c. Add an additional 1–2 reactions for pipetting error margin.
    Multiply volumes accordingly.
  d. dPCR Master Mix: 3 μL × [# reactions].
  e. ITR Mix: 0.3 μL × [# reactions].
  f. Water: 10.2 μL × [# reactions].
    Example: 13.5 μL Master Mix + 1.5 μL of 1:10K AAV dilution → Final 1:100K dilution; 13.5 μL Master Mix + 1.5 μL of 1:100K AAV dilution → Final 1:1M dilution; 13.5 μL Master Mix + 1.5 μL water → NTC.

#### Final reaction assembly

**Timing: 15 min**

25. To each sterile PCR tube, add:
  a. 13.5 μL of prepared Master Mix.
  b. 1.5 μL of diluted AAV or MilliQ water for NTC.
  c. Mix gently by pipetting.

***Note:*** Vortex each AAV dilution before pipetting. Keep all tubes on ice and protected from light throughout.

26. Load 15 μL of each final mixture into the designated wells of a QIAcuity 8.5k 24-well Nanoplate as per layout below.

**CRITICAL:** Avoid bubbles during loading.

27. Seal the nanoplate and load into the QIAcuity instrument.

**Table:** Example plate layout

Row	Column 1	Column 2	Column 3
A	AAV#1 1:100K	AAV#3 1:100K	Blank
B	AAV#1 1:100K	AAV#3 1:100K	Blank
C	AAV#1 1:1M	AAV#3 1:1M	Blank
D	AAV#1 1:1M	AAV#3 1:1M	Blank
E	AAV#2 1:100K	NTC	Blank
F	AAV#2 1:100K	NTC	Blank
G	AAV#2 1:1M	Blank	Blank
H	AAV#2 1:1M	Blank	Blank

28. Run the PCR with the cycling conditions and imaging parameters outlined below:

**Table:** PCR cycling conditions

Step	Temperature	Duration
PCR Initial Activation	95°C	2 min
2-step cycling (40 cycles)		
Denaturation	95°C	15 s
Annealing/Extension	60°C	30 s

**Table:** Imaging parameters

Channel	Exposure duration	Gain
Appropriate channel	500 ms	6

***Note:*** 1. Ensure partitions are evenly distributed and the plate is properly seated in the instrument before initiating the run.

***Note:*** 2. dPCR-based quantification typically yields titers in the range of 1E12–5E13 vg/mL, depending on vector design, production scale, and serotype.

***Note:*** 3. For applications requiring higher titers or purity, such as *in vivo* use or systemic delivery, ultracentrifugation-based purification may be used as an alternative method to isolate AAV particles and remove contaminants.

### Thawing and culture of primary human HSPCs

**Timing: ∼2.5 h**

This section has detailed steps to edit CD34^+^-enriched HSPCs isolated from umbilical cord blood, G-CSF- and/or Plerixafor-mobilized peripheral blood, and bone marrow aspirates. No modifications are made based on cell source or donor phenotype. This protocol has been applied to HSPCs from healthy donors as well as individuals with sickle cell disease,^7^^,^^8^ β-thalassemia,^9^^,^^10^ α-thalassemia,^11^ and polycythemia vera.^12^

29. Preparation and pre-expansion of human HSPCs for genome editing.
  a. Thaw HSPC Thaw Media and SFEM II media (both stored at −20°C) at 37°C.
    ***Note:*** SFEM II is to be used as base media for mixture of HSPC Culture Media, which should be prepared fresh.
  b. Place an empty 15 mL conical tube and warmed HSPC Thaw Media inside the biosafety cabinet.
    ***Note:*** Ensure materials for thawing cells are prepared and placed in the biosafety cabinet before removing the vial of HSPCs from liquid nitrogen storage.
  c. Remove the vial of frozen CD34^+^-enriched HSPCs from the liquid nitrogen tank.
  d. Quickly thaw the vial in a 37°C water bath in a swirling motion.
  e. Monitor closely and remove as soon as a small crystal remains. Do not over-thaw.
  f. Spray the thawed vial with 70% ethanol, ensuring the cap remains closed during this step.
  g. Wipe vial dry with a Kimwipe before placing it into the hood.
  h. Open the vial carefully inside the biosafety cabinet.
  i. Using a P1000 pipette, gently transfer the cell suspension into the sterile 15 mL conical tube already in the hood.
  j. Add 1 mL of HSPC Thaw Media to the empty vial to rinse residual cells.
  k. Gently pipette and slowly transfer the HSPC Thaw Media into the conical tube (recommend to do so with pipette tip submerged and slowly dispense the media in a swirling motion).
    **CRITICAL:** Be slow and cautious to minimize shear stress on freshly thawed HSPCs.
  l. Repeat step 29j-k once more with an additional 1 mL HSPC Thaw Media (with pipette tip submerged and slowly dispense the media in a swirling motion).
  m. Add an additional 10 mL of HSPC Thaw Media to conical tube (with pipette tip submerged and slowly dispense the media in a swirling motion).
  n. Incubate the tube at 37°C for 1 h to allow cell recovery.
  o. Prepare fresh complete HSPC Culture Media (can be done during 1 h incubation of HSPCs in HSPC Thaw Media):
    i. Thaw SFEM II media (we typically freeze in 25 mL aliquots, which should be sufficient for most HSPC editing experiments) at 37°C. Mix thoroughly.
    ii. Supplement the base medium with the appropriate cytokines and antibiotics as described above.
    iii. Mix thoroughly to ensure even distribution.
      ***Optional:*** If planning for downstream xenotransplantation experiments, then it is recommended to add UM171 (to a final concentration of 35 nM) in order to improve expansion of HSPCs with long-term engraftment potential.^13^
      ***Note:*** For cytokines and antibiotics, we recommend preparing small aliquots of all resuspended cytokines, pre-mixed in PCR tubes, and storage at −80°C. This allows for single-use thawing and direct addition to 25 mL of culture media to achieve the desired final concentrations. Once prepared, store the supplemented medium at 4°C for up to 1 week. Do not exceed this duration. Do not freeze aliquots of HSPC Culture Media; instead make fresh for each new experiment.
  p. After the 1-h incubation, centrifuge cells at 300 × *g* for 5 min and aspirate the HSPC Thaw Media without disturbing the cell pellet.
  q. Gently resuspend the cells in 1–2 mL of HSPC Culture Media.
  r. Count viable cells using hemocytometer or automated cell counter.
  s. Plate cells at 1–2.5 × 10^5^ cells/mL in HSPC Culture Media in T25 or T75 flasks (as per the recommended volume).
    ***Note:*** Maintaining HSPCs at 1 × 10^5^ cells/mL typically yields better proliferation (and thus improved HDR^14^) or higher densities up to 5 × 10^5^ cells/mL may be used to conserve HSPC media.
    ***Optional:*** Use flow cytometry to check viability and CD34^+^ expression.
  t. Maintain cell density at 1–5 × 10^5^ cells/mL.
  u. Incubate cultures at 37°C in a humidified incubator with 5% CO_2_.
    ***Note:* 1.** Monitor cell growth daily. Refresh media and adjust density as needed to support optimal proliferation and viability.
    ***Note:* 2.** If performing HSPC xenotransplantation experiments, then we also recommend maintaining cells at 5% O_2_.
    **CRITICAL:** Perform all steps inside a biosafety cabinet using strict aseptic technique to prevent contamination.

### CRISPR/AAV-mediated HDR

**Timing: 2–3 days (including cell expansion); ∼4 h (editing procedure)**

This section details the preparation, electroporation, and post-electroporation handling of primary CD34^+^ HSPCs with Cas9 protein (ideally high-fidelity version^15^) and gRNA and using the 4D Lonza Nucleofector. This ensures efficient delivery of ribonucleoprotein (RNP) complexes and AAV transduction for custom genome editing workflows.

#### Pre-electroporation preparation and calculations

**Timing: 1–2 h**

30. Thaw and plate CD34^+^ HSPCs 48–72 h before editing at 1 × 10^5^ cells/mL in HSPC Culture Media to allow recovery from thawing process and cell expansion.
  Perform calculations (appropriate for editing between 5 × 10^4^ and 4 × 10^6^ HSPCs per condition in a single well of a 12-well Lonza cuvette strip).
  a. gRNA:
    i. Upon receiving desiccated gRNA from vendor, reconstitute in appropriate amount of nuclease-free water (typically 30 μL for 1.5 nmol aliquot) and measure concentration on NanoDrop. Recommend to keep concentration below 4 μg/μL; if any precipitate is observed, further dilute the sample to ensure complete solubility.
    ii. Use 3.2 μg gRNA per condition.
  b. Cas9 protein:
    i. Use 6 μg per condition (e.g., stock concentration is typically 10 μg/μL, thus volume needed = 0.6 μL Cas9 per condition).
  c. P3 Electroporation Buffer (with provided Lonza supplement added):
    i. Volume = 20 μL/condition.
      ***Optional:*** AZD7648 small molecule HDR enhancer^16^: Final concentration = 0.5 μM from 2 mM stock (4,000×) (e.g., add 1.4 μL AZD7648 per 5 mL media).
  d. AAV:
    i. 5 × 10^3^ viral genomes per cell is the recommended amount of AAV to add to HSPCs, however titrations can be performed to define the amount that best balances cell viability with HDR frequency (if optimizing HDR in other cell types, then it may be appropriate to test several AAV titrations). Calculate based on AAV titer.
      ***Note:*** The amount of Cas9 and gRNA is constant regardless of whether you are editing 50K or 4M cells (calculations above are for a single well of a Lonza 12-well electroporation strip). However, the amount of AAV added is calculated directly by multiplying 5 × 10^3^ viral genomes by the number of cells. Finally, this protocol may be adapted to edit as few as 2 × 10^4^ cells in a well of a cuvette strip, although this increases the possibility of cell loss and/or decreased editing frequency. In addition, if editing >4 × 10^6^ cells, then up to 20M may be edited in a single cuvette using the same Lonza 4D machine.^17^ In that case, simply multiply all volumes of P3 buffer, Cas9, and gRNA by 5-fold (i.e., a single cuvette can be deployed using 100 μL P3 buffer, 30 μg Cas9, and 16 μg gRNA). Cuvette volume (∼100 μL) imposes a practical limit and scaling beyond this is best achieved via parallel electroporations to preserve consistency and yield.

#### Nucleofector setup

**Timing: 15–30 min**

31. Prepare the 4D Lonza Nucleofector.
  a. Mark and orient strip wells (larger hole at the top).
  b. Select DZ-100 program.
  c. Ensure correct wells and P3 buffer is selected.

***Note:*** Delivery to other cell types will require optimization of electroporation program as this has a significant impact on delivery efficiency and cell viability post-electroporation.

#### RNP complex formation

**Timing: 10–30 min**

32. Mix gRNA and Cas9 protein to form active RNP complexes before electroporation.
  a. Prepare RNP in a mini-PCR tube.
  b. Vortex and quick-spin tube of gRNA (again ensure no precipitate is formed, otherwise gRNA may need to be more thoroughly mixed or diluted).
  c. Gently flick and quick-spin tube of Cas9 protein.
  d. Add gRNA first, followed by Cas9 (as Cas9 is more viscous).
  e. Mix to ensure 3.2 μg gRNA and 0.6 μg Cas9 per reaction.
  f. Incubate Cas9:gRNA formulation at 20°C–25°C for at least 10 min.

***Note:*** Additional incubation does not improve complex formation, however, RNP complexes are stable for up to 30–60 min at 20°C–25°C.

#### Electroporation of primary HSPCs

**Timing: ∼30 min**

**Timing: 5–10 min (for step 35)**

33. Deliver RNP complexes into HSPCs using the Lonza 4D Nucleofector system.
  a. Spin down HSPCs in 15 mL conical tube and remove media.
  b. Resuspend in P3 buffer (plus supplement) at 20 μL/condition.
  c. Transfer 20 μL of cell suspension into each RNP tube, mix gently.
  d. Transfer full volume to designated strip wells.
34. Electroporate using DZ-100 pulse code.
  a. Click “Start” once plate is loaded and settings are confirmed.
  b. Confirm successful electroporation by the green plus once complete.
35. Post-electroporation rescue & culture.
  a. Following electroporation, add 80 μL warmed HSPC Culture Media directly into each well.
  b. Gently pipette to mix, then transfer to culture plate.
  c. Plate at 1–2.5 × 10^5^ cells/mL in HSPC Culture Media.
    ***Note:*** Choose appropriate plate format based on volume: e.g., 6-well plate: 2 mL of media per condition; 12-well plate: 1 mL of media per condition; 24-well plate: 0.5 mL of media per condition.
  d. Add AAV to edited cells at 5 × 10^3^ viral genomes/cell.
  e. Add AZD7648 supplement to media to boost HDR efficiency.
  f. Gently swirl to mix AAV, AZD7648, and HSPCs.
  g. Incubate cells for 48–72 h at 37°C, 5% CO_2_ in a humidified incubator.

#### Electroporation strip cleanup

**Timing: 10 min**

36. Clean strips:
  a. Spray with 70% ethanol, blot dry on Kimwipe.
  b. UV treat for at least 10 min in biosafety cabinet.
  c. Mark used wells clearly before reuse.

***Note:* 1.** Cleaned wells may be used up to 2 additional times without apparent reductions in editing frequency or cell viability.

***Note:* 2.** For transplantation studies, allow HSPCs to recover for at least 24 h. Then count cells, spin down, and resuspend in PBS containing 2% FBS. HSPCs may then be injected intravenously into immunodeficient mice (following irradiation or conditioning). A minimum of 1–5 × 10^5^ viable CD34^+^ HSPCs is recommended per mouse. Ensure that cells are kept on ice and used promptly to preserve viability and engraftment potential.

37. If cryopreserving for later use:
  a. Allow HSPCs to recover for at least 24 h prior to freezing for later use or downstream applications (including xenotransplantation).
  b. Spin down HSPCs at 300 × *g* for 5 min and aspirate media.
  c. Resuspend the cells in freezing medium (e.g., typically either Bambanker or 90% FBS + 10% DMSO) at 1.0 × 10^6^ cells/mL.
  d. Aliquot into cryovials and place in a controlled-rate freezing container at −80°C for 8–12 h, followed by long-term storage in liquid nitrogen.

### Evaluation of editing frequency

**Timing: 1–3 days (depending on method and downstream processing), as the majority of editing occurs within 72 h post-RNP electroporation and AAV transduction**^14^

This section describes the evaluation of genome editing efficiency via gDNA extraction, PCR amplification, gel purification, and sequencing.

***Note:* 1.** Standard PCR surrounding the expected cut site enables quantification of indel formation frequency. While in-out PCR (i.e., one primer located on knock-in cassette and one primer located outside homology arms in the genome (must be outside the homology arm, otherwise the PCR will primarily amplify AAV genomes)) is able to determine that some degree of editing did occur, it is not quantitative and cannot determine knock-in frequencies.

***Note:* 2.** To determine knock-in frequencies, a custom dPCR assay must be performed, typically comparing amplification of an in-out amplicon vs. a genomic DNA reference amplicon. The dPCR assay described is intended to measure the mean allelic editing frequency among a population of edited human primary HSPCs. However, if a clonal population is obtained, then the dPCR assay described will be able to determine whether the edit is homozygous or heterozygous simply by comparison to the reference gene, which will be present as 2 copies per cell. We therefore expect that the HDR allele compared to reference gene in a clonal population will be 1:1 for homozygous clones, 1:2 for heterozygous clones, and 0:1 for cells that did not undergo HDR. Alternatively, flow cytometry-based analysis may be performed to evaluate editing at the cellular (not allelic) level for edits that introduce fluorescence or modifications to surface markers.

#### Genomic DNA extraction

**Timing: 1 h**

This step outlines two options for gDNA extraction: QuickExtract method for rapid downstream use or column-based purification for higher purity of gDNA.

38. Using QuickExtract DNA Extraction Solution.
  a. Harvest ≥2–5 × 10^4^ HSPCs post-editing (2–3 days).
  b. Spin at 300 × *g* for 5 min. Aspirate media, leaving a visible pellet.
  c. If using frozen cell pellets, thaw at 20°C–25°C.
  d. Add QE buffer.
  e. Transfer to mini-PCR tubes.
    Example calculation for amount of QE buffer:
    i. 10 μL if pellet not visible.
    ii. 20 μL if pellet is visible.
    iii. 50+ μL for dense pellets.
  f. Thermocycler program:

**Table:** 

Temperature	Time
65°C	6 min
100°C	10 min
12°C	Infinite hold

The sample is ready for downstream PCR amplification using custom primer pairs.

39. Alternatively, use a commercial column-based DNA extraction kit for higher purity (protocol below is specific to QIAGEN DNeasy Blood & Tissue Kit):
  a. Perform all centrifugations at 20°C–25°C.
  b. Resuspend tissue or cell pellets in Buffer ATL.
  c. Add Proteinase K and incubate at 56°C until lysed.
  d. Add Buffer AL and incubate at 56°C for 10 min.
  e. Add ethanol (96%–100%) and mix.
  f. Load into spin columns, centrifuge, wash with Buffer AW1 and AW2.
  g. Elute in Buffer AE (200 μL).

#### PCR amplification of target locus

**Timing: ∼2 h (including setup and thermocycling)**

40. Prepare master mix for one reaction (50 μL total volume):

**Table:** 

Component	Volume	Final concentration
Nuclease-free water	23 μL	–
2× Phusion Green PCR Master Mix	25 μL	–
Forward primer	0.5 μL	0.1–0.5 μM
Reverse primer	0.5 μL	0.1–0.5 μM
Template DNA	1 μL	10 ng/μL (although a large range may be acceptable)

  a. Calculate total number of reactions and prepare extra 10%–20% to account for pipetting error.
  b. Mix reagents (excluding DNA) to make a master mix.
  c. Aliquot mix to PCR tubes and add individual DNA templates.
41. Run PCR using appropriate primers with thermocycler program:

**Table:** 

Temperature	Time
i. 95°C	10 min
ii. 94°C	30 s
iii. 55.7°C (adjust based on primer T_m_)	30 s
iv. 72°C	Adjust based on amplicon size; 15–30 s/kb
v. Repeat steps ii–iv	×50 cycles
vi. 98°C	10 min
vii. 12°C	Infinite hold

#### Agarose gel electrophoresis and gel extraction

**Timing: 1 h (for step 42)**

**Timing: 1 h (for step 43)**

**Timing: Dependent on external sequencing turnaround (for step 44)**

**Timing: ∼2 h (excluding acquisition and analysis) (for step 45)**

42. Prepare 1% agarose gel:
  a. 150 mL 1× TAE, 1.5 g agarose, 8 μL fluorescent dye (e.g., Midori Green).
  b. Load up to 50 μL of PCR product (Phusion Green contains dye) into each well, alongside a well with 10–25 μL of DNA ladder.
  c. Run gel at 150 V for 30 min.
  d. Check progress at 15 min.
  e. Place gel on a gel visualization box, then use a clean blade to cut close to band to reduce gel volume.

***Optional:*** Gel slices may be stored in 1.5 mL tubes at 4°C for up to 24 h.

43. Purify DNA using commercial gel extraction kit (protocol below is specific to QIAGEN QIAquick).
  a. Weigh gel slice; add 3× volume of Buffer QG (6× for >2% gels).
  b. Incubate at 50°C for 10 min, vortex every 2–3 min.
  c. Confirm yellow color (add sodium acetate if needed).
  d. Add 1 volume isopropanol and mix.
  e. Load onto MinElute column, centrifuge at 17,900 × *g* for 1 min.
  f. Wash with Buffer QG, then Buffer PE.
  g. Dry column with additional spin at 17,900 × *g* for 1 min.
  h. Add 10 μL Buffer EB or water directly onto membrane, incubate at 20°C–25°C for 1 min, and spin 17,900 × *g* for 1 min.

***Optional:* 1:** To maximize yield after step 43h, add another 10 μL Buffer EB or water directly onto membrane, incubate at 20°C–25°C for 1 min, and spin. To maximize concentration and increase yield, take flow through and add back to membrane, incubate at 20°C–25°C for 1 min, and spin again.

***Optional:* 2:** Confirm cleanup by running an aliquot on gel (5:1 DNA:loading dye).

44. Sanger sequencing.

***Note:*** Sanger sequencing is considered accurate down to 5% editing frequency; for lower editing frequencies, next-generation sequencing of amplicons is recommended.

45. Flow cytometry analysis (if edit introduces fluorescence or modifications to surface markers).
  a. Spin down ≥5 × 10^4^ HSPCs at 300 × *g* for 5 min.
  b. Aspirate media and resuspend pellet in 100 μL Flow Cytometry Buffer (PBS + 5% FBS + 2 mM EDTA).
  c. Stain with fluorescent antibodies (using manufacturer-recommended concentrations).
  d. Incubate at 4°C for 30 min in the dark.
  e. Wash with 1 mL Flow Cytometry Buffer and spin at 300 × *g* for 5 min.
  f. Aspirate supernatant, resuspend in 100 μL Flow Cytometry Buffer and add 1 μL of GhostRed viability dye.

#### Quantify allelic editing frequency by dPCR

**Timing: 30 min (for step 46)**

**Timing: 20 min (for step 47)**

**Timing: 2 h (for step 48)**

46. gDNA preparation and fragmentation.
  ***Note:*** Accurate dPCR analysis requires even DNA distribution across partitions. Fragmenting gDNA by restriction enzyme digestion improves uniformity.
  a. Isolate high-quality genomic DNA from HSPCs pellets using QE or column-based purification (both described above).
  b. Digest 100–500 ng of gDNA using a restriction enzyme that does not cut within the target amplicon. Perform incubation according to manufacturer’s recommendations.
47. Reaction Setup.
  a. Thaw QIAcuity Probe PCR Master Mix, primer/probe mixes, restriction enzyme-digested template DNA, and nuclease-free water.
  b. Vortex and spin down all components.
  c. Prepare the master mix according to the following guidelines:
    Example calculation (one reaction):

**Table:** 

Component	Volume	Final concentration
4× QIAcuity Probe PCR Mix	3.0 μL	1×
10× primer-probe mix (WT; e.g., *CCRL2*^18^)	1.2 μL	0.8 μM of each primer, 0.4 μM probe
10× primer-probe mix (Edited; custom designed using guidelines in before you begin section)	1.2 μL	0.8 μM of each primer, 0.4 μM probe
DNase/RNase-free water	Variable	-
Template DNA (added later)	Variable	-
*Total*	15.0 μL
  d. Vortex and spin down the PCR tubes.
  e. Dispense 12 μL of reaction mix into PCR tubes or plates.
  f. Add template DNA (volume typically 1–3 μL) to each reaction. Final reaction volume should remain at 15 μL.
  g. Gently mix by pipetting and keep tubes sealed.
  h. Transfer the final 15 μL reaction volume into the designated wells of a QIAcuity 8.5k 24-well Nanoplate.
  i. Seal the nanoplate with the provided optical film.
    **CRITICAL:** Avoid introducing air bubbles during transfer.
  j. Load the sealed nanoplate into the QIAcuity instrument.
48. Begin with the cycling parameters below.

***Note:*** Modifications may be needed to achieve optimal amplification efficiency and quantification.

**Table:** dPCR cycling conditions

Step	Temperature	Duration
PCR activation	95°C	2 min
2-step cycling (40 cycles)		
Denaturation	95°C	15 s
Annealing/extension	60°C	30 s

**Table:** Imaging parameters

Channel	Exposure duration	Gain
Appropriate channel	500 ms	6

***Note:*** Use non-fluorescent quenchers for probes. Ensure no cross-reactivity in multiplex channels.

### *In vitro* erythroid differentiation

**Timing: 14 days**

This section enables erythroid differentiation of primary human HSPCs through three defined media phases over a 14-day period. Each phase promotes sequential lineage commitment, proliferation, and terminal differentiation into erythroid cells. Established stages of HSPC-to-erythrocyte development shown below.

**Figure:**
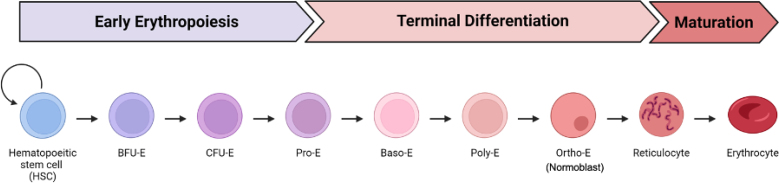

#### Phase 1–3 erythroid media preparation

49. Phase 1: Initiation of differentiation (Days 0–6).
  a. Thaw, expand, and edit HSPCs as described in the sections above.
  b. On Day 0, seed HSPCs into freshly prepared Phase 1 Erythroid Differentiation Media at a density of 1 × 10^5^ cells/mL at 37°C, 5% CO_2_, humidified incubator.
    ***Note:*** This seeding density provides an optimal balance between cellular proliferation and media usage. Higher seeding densities (2.5–5 × 10^5^ cells/mL) still mediate erythroid differentiation, though perhaps with decreased proliferation and final yield.
  c. On Day 4, quantify cell count and viability using an automated cell counter or hemocytometer.
  d. By this time, cells have typically expanded several-fold to achieve a density well above the original 1 × 10^5^ cells/mL.
  e. Dilute the culture back to 1 × 10^5^ cells/mL using Phase 1 Erythroid Differentiation Media.
  f. Transfer cells into larger tissue culture flasks or plates to accommodate increased volume, maintaining sterility.
  g. Incubate cells until Day 7 without media change.
    ***Note:*** Based on experimental needs, decide whether to carry forward the entire culture or only a subset. If fewer erythroid cells are needed downstream, a portion of the cells may be discarded.
50. Phase 2: Continued differentiation (Days 7–10).
  a. On Day 7, count cells again, as above.
  b. Centrifuge the culture at 300 × *g* for 5 min and aspirate Phase 1 media.
  c. Resuspend the cell pellet at 1 × 10^5^ cells/mL in Phase 2 Erythroid Differentiation Media.
  d. Incubate cells until day 11 without media change.

**CRITICAL:** Regularly monitor the cells under the microscope to ensure optimal cell morphology and proliferation.

51. Phase 3: Terminal maturation (Days 11–14).
  a. On Day 11, count the cells again, as above.
  b. Adjust the cell density to 1 × 10^6^ cells/mL in Phase 3 Erythroid Differentiation Media.
  c. Incubate cells until Day 14 without media change.
52. Harvest and analysis (Day 14).
  a. On Day 14, collect and count total cells.
  b. Visual inspection: A distinct red pellet indicates successful erythroid differentiation.
53. Assess differentiation efficiency via flow cytometry:

***Note:* 1.** Suggested markers: CD34, CD45, CD71, and CD235a (GPA). Additionally, CD36 may be used to mark erythroid progenitor cells, and DRAQ5 may be used to distinguish between nucleated and enucleated erythroid cell populations. Successfully differentiated erythroid cells are CD34^-^/CD45^-^/GPA^+^/CD71^+^.

***Note:* 2.** For surface markers (CD34, CD45, CD71, CD235a, and CD36), we recommend a dilution of 1:50 (e.g., 2 μL antibody per 100 μL of cell suspension in Flow Cytometry Buffer), unless otherwise specified by the manufacturer (typical stock concentration: 0.2 mg/mL). For DRAQ5, recommended dilutions range from 1:200 to 1:1,000. It is further recommended that all antibodies be titrated to determine the optimal dilution for specific experimental conditions.

54. Post-differentiation handling and downstream applications of erythroid cells.
  Upon completion of erythroid differentiation, terminally differentiated erythroid cells may be processed for various downstream applications depending on experimental needs. Below are guidelines for handling and preserving cells for different analyses.
  a. **High-performance liquid chromatography (HPLC):** To evaluate hemoglobin tetramer and/or single globin chain composition, collect a minimum of 5 × 10^5^ Day 14-differentiated erythroid cells. Pellet cells by centrifugation at 300 × *g* for 5 min and carefully aspirate the supernatant. Store cell pellet at −80°C until analysis.
  b. **RNA-sequencing (RNA-Seq):** To perform bulk RNA-Seq analysis, collect a minimum of 5.0 × 10^5^ Day 14-differentiated erythroid cells. These cells may be processed immediately using commercial RNA extraction kits or preserved in TRIzol reagent or equivalent RNA stabilization agents for future processing. This cell number typically yields sufficient RNA for downstream bulk RNA-Seq.
    ***Note:*** For TRIzol preservation: We recommend adding 1 mL of TRIzol Reagent per 5–10 × 10^6^ cells. For smaller inputs, the volume of TRIzol should be scaled accordingly, ensuring the sample volume does not exceed 25% of the TRIzol volume used. For example, 100 μL TRIzol may be used for approximately 5 × 10^5^ cells. This ratio ensures effective lysis and RNA preservation.
  c. **Cresyl blue staining:** To assess enucleation and reticulocyte maturation, collect 1–2 × 10^5^ Day 14-differentiated erythroid cells. Resuspend the cells in a 1% cresyl blue solution in PBS and incubate at 20°C–25°C for 15–30 min. Prepare cytospin slides and image under brightfield microscope to evaluate cytoplasmic and nuclear morphology. Positive staining confirms reticulocyte features.

## Expected outcomes

**Figure 1:**
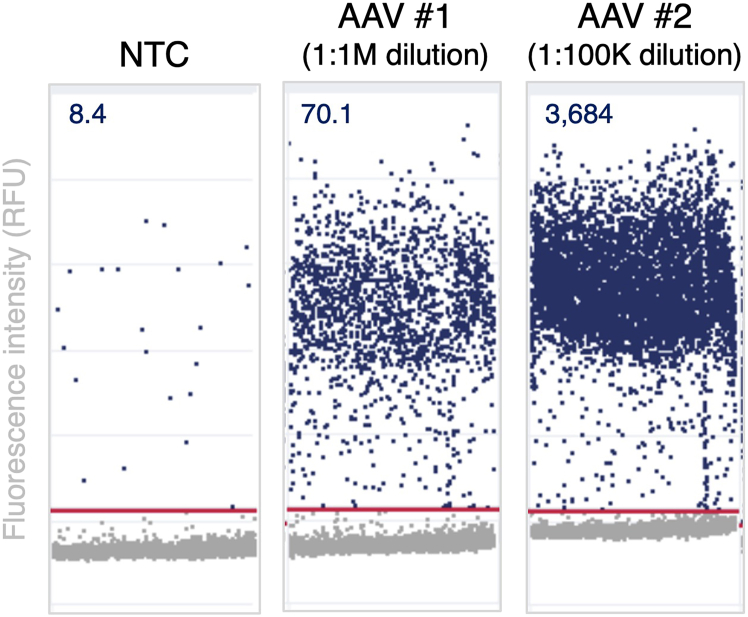
Quantification of AAV titer using dPCR Representative scatterplots showing probe fluorescence intensity for NTC sample and two different AAVs at either 1:100K or 1:1M dilutions. Scatterplots generated by QIAcuity software; numbers indicate # positive droplets / μL.

This protocol enables efficient and reproducible genome editing of primary human HSPCs using Cas9 RNP complexes and AAV-mediated HDR, with robust outcomes at each stage of the workflow. The first expected outcome following AAV production and purification is to yield high-quality AAV within the expected range of 10^8^–10^9^ vector genomes/μL following purification, depending on transfection efficiency and cell health. Quantification of AAV vector genome titers by dPCR should yield 2D scatterplots with distinct separation between positive and negative partitions, indicating high assay sensitivity and specificity. No template control (NTC) wells should exhibit minimal background signal. Accurate AAV titers are computed using the following example formula: sample yielding 3,684 positive droplets at a 1:1M dilution = 3.684 × 10^8^ AAV viral genomes/μL (Figure 1).

**Figure 2:**
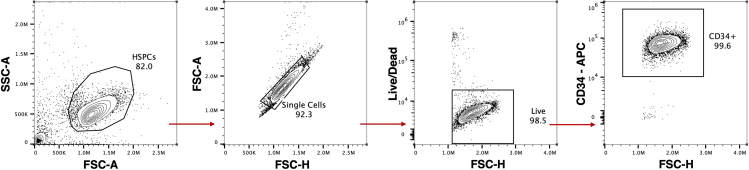
Analysis of HSPCs post-thaw using flow cytometry Representative flow cytometry plots quantify viability and CD34 expression among primary CD34-enriched HSPCs post-thaw. Percentages indicate the proportion of cells within each gate; arrows indicate that only gated cells are displayed on the subsequent plot.

Following thawing, primary human CD34^+^ HSPCs should achieve >70% viability within the first 24 h. When cultured in optimized HSPC media, cells are expected to expand several-fold over the next 48–72 h, with viability typically increasing to >90% as well as >90% CD34^+^ cells (Figure 2). These culture conditions support robust cell proliferation and maintain the potential for genome editing or erythroid differentiation.

**Figure 3:**
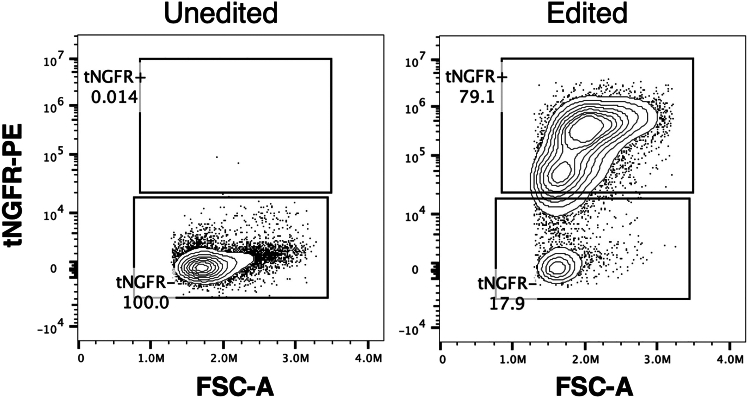
Analysis of HSPCs post-editing using flow cytometry Representative flow cytometry plots quantify viability and tNGFR marker expression at 7 days post-editing of CD34^+^ HSPCs. Percentages indicate the proportion of cells within each gate.

**Figure 4:**
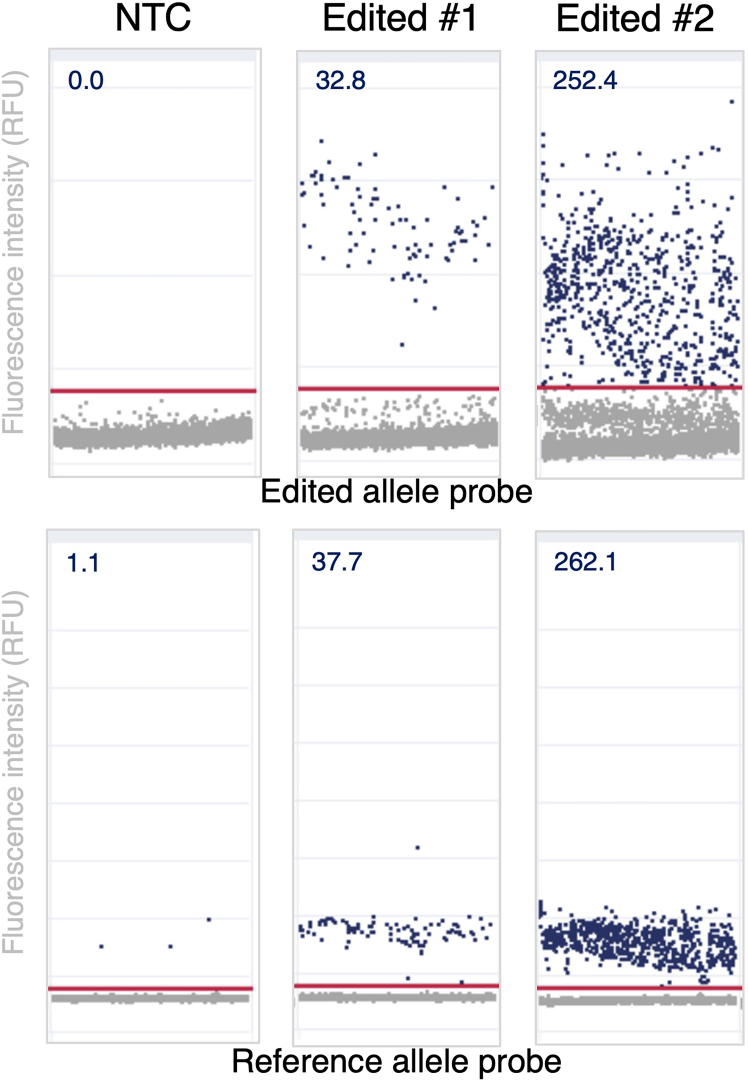
Quantification of genome editing using dPCR Representative scatterplots showing probe fluorescence intensity for NTC sample and gDNA of HSPCs 5 days post-editing. Scatterplots generated by QIAcuity software; numbers indicate # positive droplets / μL.

Expected editing frequencies can range widely depending on cleavage efficiency of the gRNA, size of the knock-in cassette, and ease of HDR at the selected locus. If selecting an effective gRNA (with cleavage efficiency >75% at the allelic level), then a frequency of HDR around 20%–60% at the allelic level can be expected. If genome editing introduces detectable markers—such as fluorescent proteins or selectable surface receptors (e.g., tCD19, tEGFR, tNGFR)—editing frequency can be measured by flow cytometry. In these cases, a clear edited vs. unedited population should emerge, enabling quantification of editing efficiency at the single-cell level (Figure 3). For precise allelic quantification, dPCR assays should again demonstrate clear partitioning and minimal NTC signal. Editing frequency is calculated as the ratio of edited to total (edited + reference) allele-positive droplets (e.g., 32.8 edited vs. 37.7 reference allele droplets/μL = 87.0% HDR) (Figure 4).

**Figure 5:**
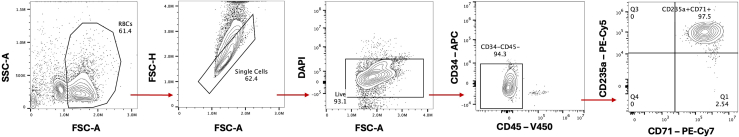
Analysis of HSPCs post-erythroid differentiation using flow cytometry Representative flow cytometry plots quantify efficiency of erythroid differentiation at Day 14 of *in vitro* HSPC-to-erythroid cell differentiation. Percentages indicate the proportion of cells within each gate; arrows indicate that only gated cells are displayed on the subsequent plot.

*In vitro* erythroid differentiation of genome-edited HSPCs over a 14-day period should yield highly viable and phenotypically mature erythroid cells. By Day 14, >90% viability is expected, with the majority of cells expressing CD71 and CD235a (GPA) and lacking CD34 and CD45, consistent with terminal erythroid maturation. This phenotype (CD34^-^/CD45^-^/CD71^+^/GPA^+^) confirms successful lineage commitment and differentiation (Figure 5), enabling downstream applications such as hemoglobin analysis, RNA-Seq, or enucleation assessment.

Together, these outcomes demonstrate the utility of the protocol for achieving high-efficiency genome editing and functional erythroid output from primary human HSPCs.

## Limitations

A major limitation in achieving high-efficiency CRISPR-Cas9-mediated HDR using AAV vectors is the identification of highly effective gRNAs. Certain genomic regions present challenges in identifying gRNAs with robust cleavage efficiency, which can significantly impair downstream knock-in success.

The maximum size of the donor sequence that can be packaged into AAV is constrained by the vector’s packaging capacity. Including the required ITRs, the AAV genome must remain below 4.5 kilobases, which restricts the size of the gene or cassette that can be knocked in. Larger genetic elements may require alternative delivery platforms.

AAV serotype tropism introduces another important limitation. While AAV6 demonstrates the highest efficiency for transduction and knock-in in primary human HSPCs, other cell types may require different serotypes for maximum transduction efficiency.

## Troubleshooting

### Problem 1

HEK 293T cells exhibit poor attachment or clumping post-thawing. Step 1.

### Potential solution

Ensure the use of low-passage cells (<15 passages) and do not exceed 80% confluency during maintenance. Avoid over-thawing and perform gentle centrifugation at 150 × *g* for 10 min and resuspension to reduce cellular stress. Discard cultures showing fused or granular morphology.

### Problem 2

Low AAV yield. Step 3.

### Potential solution

Verify the health and confluency of cells at the time of transfection (ideally 90%). Prepare fresh PEI transfection mix and incubate at 20°C–25°C for 15 min before addition. Ensure that plasmids are endotoxin-free and thoroughly dissolved without precipitate.

### Problem 3

High cytotoxicity post-transfection of AAV production plasmids. Step 3.

### Potential solution

Reduce PEI concentration if excessive cell death is observed. Alternatively, include a media change at 12–18 h post-transfection. Consider addition of alternative AAV enhancers if persistent toxicity occurs. Ensure that the transgene being packaged is not toxic to the cells.

### Problem 4

Poor recovery of AAV particles after harvest. Step 8.

### Potential solution

Ensure cells are thoroughly dislodged by mechanical scraping and that the entire volume of cells with the media is collected, including floating cells. Do not discard supernatant prematurely. Use low-retention tips and pre-chilled centrifugation conditions.

### Problem 5

Inconsistent AAV titers by dPCR. Step 23 and 24.

### Potential solution

Use validated ITR-specific primers and probes. Ensure no residual DNA contaminants in the viral prep by using benzonase digestion. Always prepare dPCR master mix fresh, vortex reaction mixes and dilutions thoroughly, and minimize freeze-thaw cycles of primer/probe stocks.

### Problem 6

Low viability of HSPCs post-thaw. Step 29

### Potential solution

Rapidly thaw cells in a swirling motion and add pre-warmed HSPC Thaw Media in a gradual, dropwise manner to reduce osmotic shock. Avoid over-thawing and perform gentle centrifugation at 150 × *g* for 10 min and resuspension to reduce cellular stress. Minimize mechanical stress during pipetting.

### Problem 7

Low HDR frequency in HSPCs. Step 30.

### Potential solution

First verify gRNA editing efficiency at the intended knock in site by PCR and Sanger sequencing. If low/no indel formation, then the problem likely lies with the Cas9 protein, gRNA, electroporation buffer, and/or execution of electroporation program. However, if indel formation is observed, then the problem likely lies with AAV transduction and/or downstream cell culture. To optimize, maintain cell density between 1.0–2.5 × 10^5^ cells/mL post-electroporation. Use freshly prepared RNP complexes and high-titer AAV stocks. Ensure accurate Cas9:gRNA stoichiometry and appropriate AZD7648 supplementation. Avoid delays between electroporation and cell rescue as extended time in chilled electroporation mix reduces cell viability.

## Resource availability

### Lead contact

Further information and requests for resources and reagents should be directed to and will be fulfilled by the lead contact, Kyle Cromer (kyle.cromer@uscf.edu).

### Technical contact

Questions about the technical specifics of performing the protocol should be directed to the technical contacts, Devesh Sharma and Roshani Sinha (devesh.sharma@uscf.edu & roshani.sinha@ucsf.edu).

### Materials availability

This study did not generate new unique reagents.

### Data and code availability

This study did not generate datasets or code.

## Acknowledgments

The authors thank the following funding sources that made this work possible: B.J.L. was supported by the 10.13039/100000001National Science Foundation Graduate Research Fellowship Program; M.K.C. was supported by the 10.13039/100031826American Society of Gene & Cell Therapy Career Development Award and the 10.13039/100000002National Institutes of Health (R01-HL161291).

## Author contributions

D.S., R.S., B.J.L., and M.K.C. designed experiments. D.S., R.S., and B.J.L. carried out experiments. D.S., R.S., B.J.L., and M.K.C. analyzed data. D.S., R.S., and M.K.C. wrote the manuscript.

## Declaration of interests

M.K.C. has equity in Kamau Therapeutics. M.K.C. holds patents related to this work (WO/2023/060059, WO 2023/028469, WO 2023/060059, WO 2023/064798, WO 2021/097350, and WO/2021/022189).

## Declaration of generative AI and AI-assisted technologies in the writing process

During the preparation of this work, the authors used ChatGPT-4 by OpenAI, Inc. solely to improve language and readability. After using these tools, the authors reviewed and edited the content as needed and take full responsibility for the content of the publication.
